# Comparative Proteomic Analysis of Human Cholangiocarcinoma Cell Lines: S100A2 as a Potential Candidate Protein Inducer of Invasion

**DOI:** 10.1155/2015/629367

**Published:** 2015-04-27

**Authors:** Kasima Wasuworawong, Sittiruk Roytrakul, Atchara Paemanee, Kattaleeya Jindapornprasert, Waraporn Komyod

**Affiliations:** ^1^Department of Biochemistry, Faculty of Science, Mahidol University, 272 Rama VI Road, Bangkok 10400, Thailand; ^2^Proteomics Research Laboratory, Genome Institute, National Science and Technology Development Agency, 111 Thailand Science Park, Phahonyothin Road, Pathum Thani 12120, Thailand

## Abstract

Cholangiocarcinoma (CCA) is a bile duct cancer, commonly found in Asia including Thailand and especially in the northeastern region of Thailand. To identify the proteins involved in carcinogenesis and metastasis of CCA, protein expression profiles of high-invasive KKU-M213 and low-invasive KKU-100 cell lines were compared using a comparative GeLC-MS/MS proteomics analysis. A total of 651 differentially expressed proteins were detected of which 27 protein candidates were identified as having functions involved in cell motility. A total of 22 proteins were significantly upregulated in KKU-M213, whereas 5 proteins were downregulated in KKU-M213. S100A2, a calcium-binding protein in S100 protein family, is upregulated in KKU-M213. S100A2 is implicated in metastasis development in several cancers. The protein expression level of S100A2 was verified by Western blot analysis. Intriguingly, high-invasive KKU-M213 cells showed higher expression of S100A2 than KKU-100 cells, consistent with proteomic data, suggesting that S100A2 may be a key protein involved in the progression of CCA. However, the biological function of S100A2 in cholangiocarcinoma remains to be elucidated. S100A2 might be a potential biomarker as well as a novel therapeutic target in CCA metastasis.

## 1. Introduction

Cholangiocarcinoma (CCA) is a malignant tumor that originates from epithelial cells of the bile duct. CCA is difficult to diagnose and the curative treatment remains challenging. Due to its late clinical manifestation, morbidity and mortality rates of CCA are high and its incidence has been increasing over the past three decades, especially in northeastern Thailand [1]. CCA is often associated with metastasis which is a highly complicated process that involves cell motility, invasion, angiogenesis, intravasation of tumor cells into the blood stream, and finally extravasation and colonization of tumor cells at secondary sites [2]. The migration and invasion properties have been a hallmark of cancer [3] including CCA, in incrimination of disease severity. In particular, metastasis is one of the major hindrances to the treatment of CCA and many cancer types that cause more than 90% of cancer-associated mortality [4]. Moreover, CCA is resistant to radio- and chemotherapy, and surgical resection is the only effective therapy against this type of cancer [5–7]. Hence, understanding the mechanism of invasion and metastasis will be important in identifying key players involved, which may lead to development of effective targeted therapy against this deadly disease.

Here, we compared the protein profiles of two human CCA cell lines with different metastatic abilities, KKU-M213 and KKU-100. KKU-M213, a high-invasive cell line, originated from adenosquamous CCA with well differentiation and KKU-100, a low-invasive cell line, was isolated from adenocarcinoma CCA with poor differentiation [8]. Studying the differential protein patterns of these cell lines allowed us to identify several proteins which might be the key determinant of the metastatic properties of the CCA cells and might be beneficial as a future drug target.

Proteomics analysis is currently considered to be a powerful tool for global evaluation of protein expression, and proteomics has been widely applied in analysis of diseases, especially in fields of cancer research. In this study, we employed a comparative SDS-PAGE coupled with LC-MS/MS (GeLC-MS/MS) based proteomics approach [9] to compare the protein expression profile of the high-invasive KKU-M213 cell line with low-invasive KKU-100 cell line to better understand the development and metastasis of CCA. MS/MS spectra of obtained proteins were identified based on NCBI human database. This technique can identify potential candidate proteins that might be involved in the different degrees of invasiveness displayed by the two CCA cell lines. Differential expression at transcription and protein expression levels of a candidate protein was further confirmed by quantitative real-time PCR and Western blot analysis.

## 2. Materials and Methods

### 2.1. Cell Culture

Human cholangiocarcinoma cell lines, KKU-M213 and KKU-100, were kindly provided by Professor Banchob Sripa (Khon Kaen University, Khon Kaen, Thailand). Cells were cultured in Ham's F-12 nutrient mixture medium (Invitrogen Corp., Auckland, NZ) supplemented with 10% fetal bovine serum (FBS), 100 U/mL penicillin, 100 *μ*g/mL streptomycin sulfate (Invitrogen Corp., Auckland, NZ), and 15 mM HEPES (USB Corp., OH, USA). Cells were incubated at 37°C in a humidified atmosphere with 5% CO_2_.

### 2.2. Invasion Assay

Invasion assay was determined by Matrigel transwell* in vitro* invasion assay as previously described [10] with some modification. In brief, the upper chamber of a transwell unit (6.5 mm diameter polycarbonate membrane with 8 *μ*m pore size) (Corning Incorporated Life Science, Corning, NY) was coated with 30 *μ*g of Matrigel (BD Biosciences, Bedford, MA). Cells (80% confluent) were harvested using 0.25% Trypsin-EDTA (Invitrogen Corp., Auckland, NZ) and resuspended in serum-free media. A 200 *μ*L aliquot of cell suspension (10^5^ cells) was added to the upper chamber. The lower chamber was filled with 600 *μ*L of media containing 1% FBS. After 12 hours of incubation at 37°C under CO_2_ atmosphere, noninvading cells in the upper chamber were removed and cells that invaded the Matrigel and had attached to the lower surface of the transwell membrane were fixed with 25% methanol for 15 min and stained with 0.5% crystal violet. Invaded cells were counted in 5 random fields under light microscope at 10x magnification. The reported values represent mean ± SE of the results obtained from three independent experiments.

### 2.3. Preparation of Cell Lysates

Cells were washed twice with cold PBS containing 100 *μ*M Na_3_VO_4_, trypsinized and collected by centrifugation. Cell pellets were kept in −80°C prior to use. The pellets were lysed in Tris-lysis buffer (20 mM Tris-HCl, pH 7.5; 150 mM NaCl; 10 mM NaF; 1% (v/v) Triton-X) supplemented with 1 mM Na_3_VO_4_, 1 mM PMSF, and protease inhibitors (Sigma, UK) and chilled on ice for 30 min. Lysates were centrifuged at 13000 rpm for 10 min at 4°C to discard cell debris and protein concentration in the supernatant was determined by Lowry assay [11]. The cell lysates were stored at −20°C.

### 2.4. One-Dimension Gel Electrophoresis and Tryptic In-Gel Digestion

Cell lysates (30 *μ*g) were mixed in loading buffer (312.5 mM Tris-Cl, pH 6.8, 50% glycerol, 10% SDS, 0.05% bromophenol blue, 12.5% 2-B-mercaptoethanol) and boiled for 5 min before being applied on a 12.5% SDS-polyacrylamide gel (BioRad, Hercules, CA) using Hoefer apparatus. After Coomassie blue staining, gel slices were excised, cut into 1 mm^3^ cubes, and subjected to in-gel tryptic digestion. The excised gel slices were reduced with 10 mM DTT/10 mM NH_4_HCO_3_, alkylated with 100 mM IAA/10 mM NH_4_HCO_3_, and digested with 1 ng protein per 20 ng sequencing grade trypsin (Promega, Germany) at 37°C overnight.

### 2.5. Protein Identification Using LC-MS/MS

Tryptic peptides were protonated with 0.1% formic acid before injection into NanoAcquity system (Waters Corp., Milford, MA) equipped with a Symmetry C_18_ 5 *μ*m, 180-*μ*m × 20-mm Trap column and a BEH130 C_18_ 1.7 *μ*m, 100-*μ*m × 100-mm analytical reversed phase column (Waters Corp., Milford, MA). [Glu^1^] fibrinopeptide B was used as the reference sprayer of the NanoLockSpray source of the mass spectrometer. Analysis of tryptic peptides was performed using a SYNAPT HDMS mass spectrometer (Waters Corp., Manchester, UK). The time-of-flight analyzer of the mass spectrometer was externally calibrated with [Glu^1^] fibrinopeptide B. The quadrupole mass analyzer was adjusted such that ions from *m*/*z* 300 to 1,800 were efficiently transmitted. BSA, used for normalization, was performed along with the samples.

MS intensities of individual LC-MS analysis were differentially quantified by using DeCyder MS Differential Analysis Software (GE Healthcare, USA). PepMatch module was used for evaluating the average abundance ratio of each sample peptide, allowing for automated detection of peptides and assignment of charge states. The MS/MS data was searched against the NCBInr database and identified by using Mascot software (Matrix Science, London, UK). Database interrogation was implemented as follows: taxonomy—homo sapiens; database—NCBInr; enzyme—trypsin; fixed modification—carbamidomethyl; variable modification—oxidation of methionine residues; mass values—monoisotopic; peptide mass tolerance—2 Da; peptide charge state—1+, 2+, and 3+. Protein accession numbers were classified according to their biological function by PANTHER Classification system version 8.1 (http://www.pantherdb.org/geneListAnalysis.do).

### 2.6. Western Blot Analysis

A total of 30 *μ*g of protein lysates was separated by 12% SDS-PAGE and then transferred to PVDF membrane (Pall, Germany) by semidry electroblotting at constant voltage (25 V) for 60 min. The membranes were blocked with 5% BSA in 1x TBS-N for 1 hr and then incubated with anti-S100A2 primary antibody (Abcam, UK) at 4°C overnight. The blots were washed three times for 5 min with TBS-N buffer and incubated with anti-rabbit HRP-conjugated secondary antibody (Santa Cruz Biotechnology Inc., Santa Cruz, CA) at room temperature for 30 min. Signals were detected using Amersham ECL Prime Western Blotting Detection Reagent (GE Healthcare, USA).

### 2.7. Quantitative Real-Time PCR

Total RNA was extracted using illustra RNAspin Mini kit (GE Healthcare, USA) as described by the manufacturer. 1 *μ*g of total RNA was converted to cDNA using ImProm-II Reverse Transcription System kit (Promega, Germany) using random hexamer primers according to the manufacturer's description. The PCR reaction was performed in a final volume of 20 *μ*L containing 100 ng of cDNA, 300 nM of each primer, and 10 *μ*L FastStart Universal SYBR Master (Roche, Germany). Specific primers were as follows: S100A2 forward 5′-CTGGGTCTGTCTCTGCCACC-3′, S100A2 reverse 5′-GCAGGAGTACTTGTGGAAGGTAGTG-3′ and *β*-actin forward 5′-CTCTTCCAGCCTTCCTTCCT-3′, *β*-actin reverse 5′-AGCACTGTGTTGGCGTACAG-3′. Thermal cycling conditions were as follows: denaturing at 95°C for 10 min followed by 40 cycles of 95°C for 15 s and 60°C for 30 s. Real-time PCR was performed on Mx3000P QPCR System (Agilent Technologies, USA). All PCR amplifications were conducted in triplicate. The 2^−ΔΔCT^ method [12] was used to calculate the relative gene expression level.

### 2.8. Statistical Analysis

Statistical analysis was performed using Student's *t*-test with *P* < 0.05 considered to be significant.

## 3. Results and Discussion

**Figure 1:**
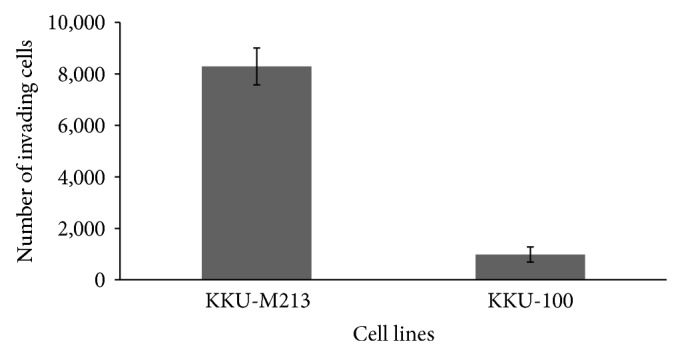
*In vitro* invasion assays of KKU-M213 and KKU-100 cells were conducted in a transwell unit coated with Matrigel. Cells in serum-free medium were plated in the upper chamber of a transwell unit. After 12 hours of incubation, cells invading to the lower compartment of the transwell unit were stained and counted. The numbers of invading cells are presented as mean ± SE of results obtained from three independent experiments, *P* < 0.01, compared with KKU-100 cell line.

**Figure 2:**
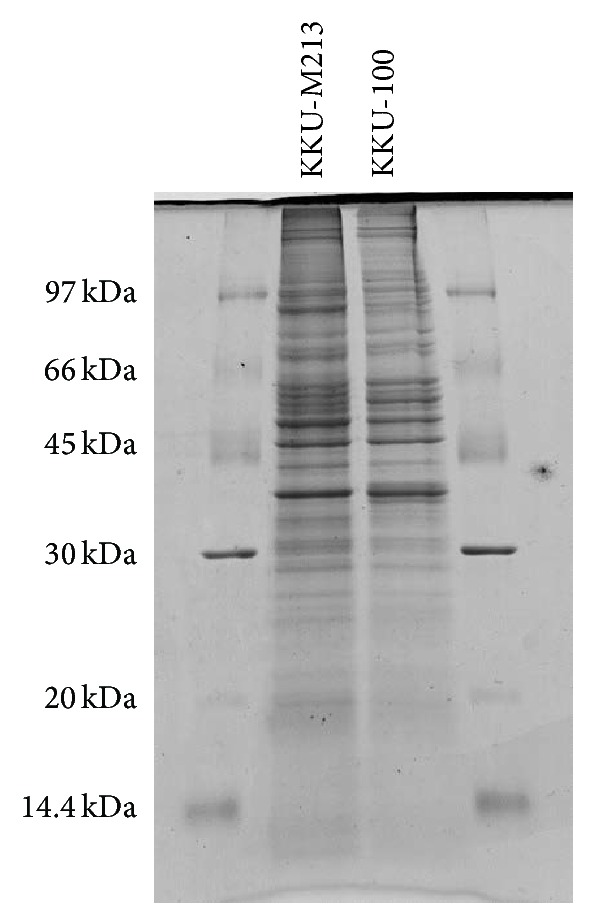
Differential expression of proteins comparing between KKU-M213 and KKU-100 cell lines. 30 *μ*g of protein lysates was separated in 12.5% SDS-PAGE with constant amplitude (20 mA/gel). The gel was stained with Coomassie blue R-250 to visualize protein bands.

**Figure 3:**
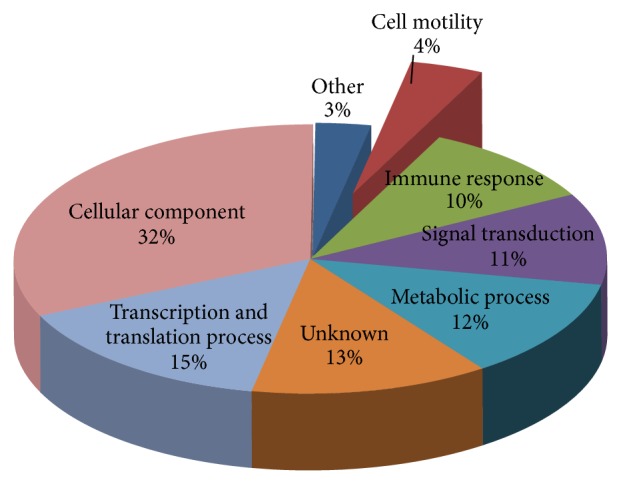
Gene ontology pie-charts showed categorization of 651 identified proteins from MS/MS spectra according to their biological processes using the PANTHER classification system.

Cell invasion using the Boyden-transwell migration assay revealed that the KKU-M213 cells displayed approximately 8-fold higher level of invasiveness than KKU-100 cells (Figure 1). The results were consistent with previous report [13] that KKU-M213 is a high-invasive cell line while KKU-100 is a low-invasive cell line. A comparative SDS-PAGE of protein lysates of both cell types initially indicated differences in intensity of protein bands as shown in Figure 2. Upon in-gel tryptic digestions coupled with LC-MS/MS (GeLC-MS/MS) analysis of the protein lysates of high-invasive KKU-M213 cells and low-invasive KKU-100 cells, six hundred and fifty-one differentially expressed proteins were identified. These proteins were classified into 8 groups of functional proteins according to their biological processes synergized by UniProtKB, using PANTHER classification system. These proteins were categorized as cellular component (32%), transcription and translation process (15%), metabolic process (12%), signal transduction (11%), immune response (10%), cell motility (4%), unknown (13%), and others (3%) (Figure 3). Among these, 27 proteins were identified belonging to the cell motility group. The relative expression of these proteins was determined by reciprocal common fraction between peptide intensities of the two cell lines. With this approach, a total of 22 proteins were found to be significantly upregulated in KKU-M213 (Table 1), whereas 5 proteins were downregulated in KKU-M213 (Table 2).

**Figure 4:**
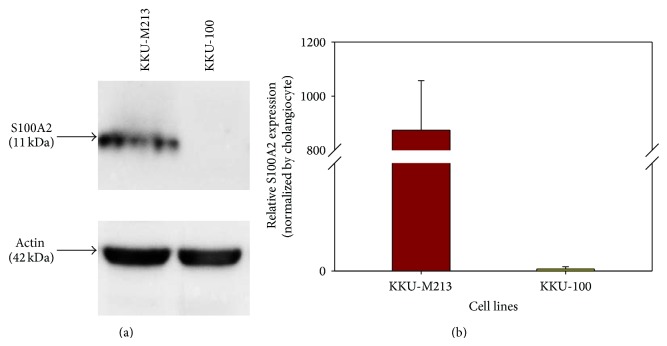
Validation of S100A2 in cholangiocarcinoma KKU-M213 and KKU-100 cell lines. (a) Western blot analysis. (b) Quantitative real-time PCR. Data are presented as mean ± SE of S100A2 mRNA level normalized with *β*-actin mRNA obtained from three independent experiments, *P* = 0.003, compared with KKU-100 cell line.

As shown in Table 1, the expression of S100A2 is notably higher in KKU-M213 than in KKU-100. The S100A2 protein is a calcium-binding protein in S100 protein family and has been implicated in the initiation and progression of human cancers such as epithelial ovarian cancer, pancreatic cancer, and gastric cancer [14, 15] and is associated with cancer metastasis process [15–17]. Further verification of the protein expression level of S100A2 by Western blot analysis using an antibody specific to S100A2 confirmed that the expression of S100A2 in KKU-M213 is obviously higher than KKU-100 cells (Figure 4(a)), in compliance with the expression profile of proteomic analysis. Moreover, S100A2 had higher expression in KKU-M213 than MMNK-1, a normal cholangiocyte cell line, since S100A2 protein expression was not detected in MMNK-1 cells (data not shown). The expression of S1000A2 at transcription level as determined by quantitative real-time PCR also elucidated about 850-fold higher expression in KKU-M213 than KKU-100 (Figure 4(b)). Altogether the expression levels of S100A2 in correlation with the invasiveness of KKU-M213 cells implied that S100A2 might be a key protein involved in the progression of CCA. S100A2 has been reported to be a potential biomarker for diagnosis and prognosis in many types of cancer [14], with overexpression and downregulation in various types of cancer [15, 18–22]. The role of S100A2 in promoting NSCLC metastasis [18] and migration/invasion in hepatocellular carcinoma [23] has also been documented. The S100A2 expression has been shown to be necessary for TGF-*β*-mediated migration/invasion [23] and was regulated by TGF-*β*-induced MEK/ERK signaling. Ectopic expression of S100A2 also elucidated that S100A2 regulated PI3K/Akt signaling, a potent pathway involved in epithelial-mesenchymal transition (EMT) [24]. Furthermore, S100A2 can interact with p53 to modulate its transcriptional activity in the calcium-dependent manner [25] while Smad3 does not need calcium ion to interact with S100A2 [24]. Taken together our findings of the S100A2 differential overexpression in KKU-M213 signified its role in invasive ability in CCA. Recently, immunohistochemistry of resected CCA tissues illustrated that S100A2 expression level was correlated with severity of CCA cancer progression and suggested it as a potential biomarker for the diagnosis of cholangiocarcinoma patients [26]. However, the biological function of S100A2 toward invasiveness and progression of cholangiocarcinoma still needs further investigations. Importantly our results suggested that S100A2 could be a candidate biological marker and novel target for diagnosis of CCA metastasis.

## 4. Conclusions

Our study aims to compare the protein profiles of the two CCA cell lines, KKU-M213 and KKU-100, with an attempt to identify proteins associated with invasiveness of CCA. SDS-PAGE coupled with LC-MS/MS (GeLC-MS/MS) is a potential initial technique to obtain an entire protein expression profile, followed by further verification steps. With this method, we showed a profile of proteome alterations in the two CCA cells with different invasive ability. We have identified 651 proteins that were found to be differentially expressed between the two cell lines and could be categorized into at least 6 functional groups including cellular component (32%), transcription and translation process (15%), metabolic process (12%), signal transduction (11%), immune response (10%), and cell motility (4%). In cell motility group, S100A2, a calcium-binding protein, which had pronouncedly 1.8-fold higher expression in high-invasive KKU-M213 cells, has been identified. Higher expression of S100A2 was confirmed at both transcription and protein expression levels. Our results suggested that S100A2 could be a significant candidate marker of CCA carcinogenesis and possibly a novel therapeutic target in CCA metastasis. Further investigation of the biological function of S100A2 in CCA could be pursued by overexpressing of S100A2 in S100A2-depleted cell line and by an approach using knock-down protein expression in S100A2-expressing cell line. Finally our observations by proteomic approach provided useful insights for understanding the mechanism involved in CCA carcinogenesis and could have implications in improved CCA diagnosis and prognosis capability.

## Figures and Tables

**Table 1:** Overexpression of proteins in KKU-M213 compared to KKU-100.

Protein	ID details	Sequence	Score	Fold^a^
gi∣5174661	Protein S100A2	ELPSFVGEK	22.51	1.80
gi∣18088719	Tubulin, beta	ISVYYNEATGGK	60.59	1.68
gi∣12667788	Myosin-9	IAQLEEQLDNETK	33.10	1.58
gi∣4885583	Rho-associated protein kinase 1	SVAMCEMEK	2.96	1.47
gi∣66346662	Rho GTPase-activating protein 8 isoform 1	KDGDLTMWPR	20.28	1.42
gi∣46249758	Ezrin	IALLEEAR	49.55	1.33
gi∣5174735	Tubulin beta-4B chain	INVYYNEATGGK	64.96	1.29
gi∣116063573	Filamin-A isoform 1	SPFEVYVDK	36.32	1.27
gi∣4502101	Annexin A1	TPAQFDADELR	93.72	1.24
gi∣33469929	Pikachurin isoform 1 precursor	QKIVEGMAEGGFTQIK	3.99	1.23
gi∣47059046	Protocadherin-23 isoform 1	AVPPRMPAVNLGQVPPK	9.20	1.23
gi∣4501891	Alpha-actinin-1 isoform b	AGTQIENIEEDFRDGLK	20.13	1.21
gi∣336020355	Mitogen-activated protein kinase 4 isoform 2	LTANETQSASSTLQK	10.91	1.21
gi∣50845388	Annexin A2 isoform 1	GVDEVTIVNILTNR	79.32	1.20
gi∣28372535	Tctex1 domain-containing protein 3	VQQILTESLK	30.94	1.20
gi∣40788018	Rho GTPase-activating protein 11A isoform 2	MSSNTEKK	8.47	1.18
gi∣105990514	Filamin-B isoform 2	VLFASQEIPASPFR	40.73	1.15
gi∣224451128	Protein eyes shut homolog isoform 1	ISDISFHYEFHLK	13.75	1.15
gi∣122937398	Cytoplasmic dynein 2 heavy chain 1 isoform 2	AADLKDLNSR	15.59	1.13
gi∣4503355	Dedicator of cytokinesis protein 1	KVTAKIDYGNR	3.12	1.10
gi∣7662284	Protein-methionine sulfoxide oxidase MICAL2	AAHLASMFGHGDFPQNK	11.55	1.08
gi∣7657532	Protein S100A6	LMEDLDR	36.48	1.08

^a^Statistical significance was determined by Student's *t*-test (*P* < 0.05).

**Table 2:** Overexpression of proteins in KKU-100 compared to KKU-M213.

Protein	ID details	Sequence	Score	Fold^a^
gi∣4504981	Galectin-1	SFVLNLGK	30.26	2.33
gi∣4506091	Mitogen-activated protein kinase 6	RLDHDNIVK	9.03	1.39
gi∣62548860	Matrilin-2 isoform a precursor	NFNSAKDMK	12.44	1.21
gi∣53828924	Neuropeptides B/W receptor type 1	TYSAAR	9.92	1.22
gi∣6005810	Mitogen-activated protein kinase 1 isoform 2	LGGGTYGEVFK	8.48	1.12

^a^Statistical significance was determined by Student's *t*-test (*P* < 0.05).
